# Association of semaglutide with reduced incidence and relapse of cannabis use disorder in real-world populations: a retrospective cohort study

**DOI:** 10.1038/s41380-024-02498-5

**Published:** 2024-03-14

**Authors:** William Wang, Nora D. Volkow, Nathan A. Berger, Pamela B. Davis, David C. Kaelber, Rong Xu

**Affiliations:** 1 Center for Science, Health, and Society, Case Western Reserve University School of Medicine, Cleveland, OH USA; 2 National Institute on Drug Abuse, National Institutes of Health, Bethesda, MD USA; 3 Center for Community Health Integration, Case Western Reserve University School of Medicine, Cleveland, OH USA; 4 Center for Clinical Informatics Research and Education, The MetroHealth System, Cleveland, OH USA; 5 Center for Artificial Intelligence in Drug Discovery, Case Western Reserve University School of Medicine, Cleveland, OH USA

**Keywords:** Addiction, Psychiatric disorders

## Abstract

Cannabis is the most frequently used illicit drug in the United States with more than 45 million users of whom one-third suffer from a cannabis use disorder (CUD). Despite its high prevalence, there are currently no FDA-approved medications for CUD. Patients treated with semaglutide, a glucagon-like peptide-1 receptor agonist (GLP-1RA) approved for treating type 2 diabetes (T2D) and for weight management have reported reduced desire to drink and smoke. Preclinical studies have shown that semaglutide decreased nicotine and alcohol consumption. Preclinical and preliminary clinical evidence of semaglutide’s potential beneficial effects on various substance use disorders led us to evaluate if it pertained to CUD. In this retrospective cohort study of electronic health records (EHRs) from the TriNetX Analytics Network, a global federated health research network of approximately 105.3 million patients from 61 large healthcare organizations in the US, we aimed to assess the associations of semaglutide with both incident and recurrent CUD diagnosis compared to non-GLP-1RA anti-obesity or anti-diabetes medications. Hazard ratio (HR) and 95% confidence intervals (CI) of incident and recurrent CUD were calculated for 12-month follow-up by comparing propensity-score matched patient cohorts. The study population included 85,223 patients with obesity who were prescribed semaglutide or non-GLP-1RA anti-obesity medications, with the findings replicated in 596,045 patients with T2D. In patients with obesity (mean age 51.3 years, 65.6% women), semaglutide compared with non-GLP-1RA anti-obesity medications was associated with lower risk for incident CUD in patients with no prior history CUD (HR: 0.56, 95% CI: 0.42–0.75), and recurrent CUD diagnosis in patients with a prior history CUD (HR: 0.62, 95% CI: 0.46–0.84). Consistent reductions were seen for patients stratified by gender, age group, race and in patients with and without T2D. Similar findings were replicated in the study population with T2D when comparing semaglutide with non-GLP-1RA anti-diabetes medications for incident CUD (HR: 0.40, 95% CI: 0.29–0.56) and recurrent CUD (HR: 0.66, 95% CI: 0.42–1.03). While these findings provide preliminary evidence of the potential benefit of semaglutide in CUD in real-world populations, further preclinical studies are warranted to understand the underlying mechanism and randomized clinical trials are needed to support its use clinically for CUD.

## Introduction

Cannabis is the most widely used illicit substance worldwide, with estimates from the United Nations Office on Drugs and Crime (UNODC) that in 2020, 209 million people or 4% of the global adult population, had used cannabis (range: 141 million–256 million) [1]. In the United States, the increased legalization of recreational and medical cannabis by the States has expanded the number of users who consume cannabis regularly and at higher doses [2], which is driven both by the availability of higher potency cannabis products [3] and access to diverse formulations (e.g., edibles, beverages, vaping, and dabbing) [4]. Indeed, in the United States between 2008 and 2019, the number of past-year cannabis users increased from 22.6 million to 45.0 million, that of daily or near-daily users increased from 3.6 to 9.8 million, and the number of adults with cannabis use disorder (CUD) increased from 3.4 to 4.1 million [5]. Nearly a quarter of cannabis users who meet the DSM-5 (the Diagnostic and Statistical Manual of Mental Disorders, Fifth Edition) criteria for CUD have a severe disorder that interferes with their everyday life [6]. Moreover, CUD has been associated with a higher risk for psychotic disorders [7, 8], self-harm, and suicidal behaviors [9–11]. Despite the high prevalence of CUD and its negative consequences, there are currently no FDA-approved medications. Treatment of CUD is managed by tapering marijuana use and providing support to combat withdrawal symptoms alongside behavioral interventions. However, behavioral therapies such as contingency management, cognitive-behavioral, and motivational enhancement therapies, are only moderately effective. Thus, there is an urgency to develop medications that can help treat CUD.

Clinical anecdotes that patients treated with semaglutide, a glucagon-like peptide-1 receptor (GLP1R) agonist approved for treating type 2 diabetes (T2D) in 2017 and for weight management in 2021 reported reduced desire to drink and smoke have attracted attention regarding its potential to treat addiction [12]. Currently, several registered clinical trials are ongoing to evaluate the effect of semaglutide on alcohol consumption [13–17] and on smoking cessation [18, 19]. Preclinical studies have investigated the effects of semaglutide or other GLP1R agonizts on nicotine, alcohol, cocaine or opioids [20–23]. However, little attention has been placed on the effects of semaglutide or other GLP1R agonizts on cannabis consumption even though the prevalence of cannabis use is even higher than that of tobacco use among certain demographics. Preclinical and preliminary clinical evidence of semaglutide’s potential beneficial effects in various substance use disorders led us to evaluate if it could extend to CUD. Here we used a large electronic health record (EHR) database to conduct a nationwide multicenter retrospective cohort study in patients who were obese with or without a pre-existing CUD and who were prescribed semaglutide vs. non-GLP-1RA anti-obesity medications to determine whether semaglutide was associated with changes in both the incidence and recurrence of CUD. Outcomes were separately assessed by age groups, sex, and race. Findings were replicated in a separate study population of patients with T2D during a non-overlapping period.

## Method

### Database

We used TriNetX, a global federated health research network providing analytics to access and analyze de-identified and aggregated electronic medical records (diagnoses, procedures, medications, laboratory values, genomic information) from approximately 105.3 million patients in 61 large healthcare organizations. These healthcare organizations cover diverse geographic regions, age, race/ethnic, income and insurance groups, and clinical setting [24]. The MetroHealth System, Cleveland OH, IRB determined research using TriNetX as described in this study was not Human Subject Research, and IRB approval is not required (more details in the Supplementary Appendix). The TriNetX platform has been used in retrospective cohort studies [25–38] including evaluating risk and health outcomes of patients with substance use disorders including CUD [25, 32, 35] and the associations of GLP-1RAs with cancer [37] and semaglutide with suicidal ideations [38].

### Study populations

#### The study populations with obesity

The analyses in patients with obesity were restricted to a starting date of 6/2021 and an ending date of 12/2022. The starting date was when semaglutide was approved in the US for weight management as Wegovy. The ending date of 12/2022 was chosen to allow for a 12-month follow-up period by the time of data analysis on January 21, 2024. To assess the associations of semaglutide with incident CUD (first time diagnosis of CUD), the study population included 83,189 patients who had active medical encounters for the diagnosis of obesity in 6/2021–12/2022, were for the first time (new users design) prescribed semaglutide or non-GLP-1RA anti-obesity medications (bupropion, naltrexone, orlistat, topiramate, phentermine) [39] during 6/2021-12/2022 (time zero or index event), had no diagnosis of CUD on or before the index event and had a diagnosis of at least one of obesity-associated comorbidities (T2D, hypertension, hypercholesterolemia, hyperlipidemia, heart diseases, stroke) on or before the index event. Patients who were prescribed other GLP-1RAs or had bariatric surgery on or before the index event were excluded. This study population was then divided into two cohorts: (1) Semaglutide cohort – 45,445 patients prescribed semaglutide, and (2) non-GLP1-RA anti-obesity medication cohort – 37,744 patients prescribed non-GLP-1RA anti-obesity medications but not semaglutide. We used new-user design to mitigate prevalent user bias and confounding associated with the drug itself [40, 41].

To assess the associations of semaglutide with recurrent CUD, the study population included 2034 patients who had active medical encounters for the diagnosis of obesity in 6/2021–12/2022, were for the first time (new users design) prescribed semaglutide or non-GLP-1RA anti-obesity medications during 6/2021–12/2022 (time zero or index event), had a diagnosis of CUD on or before the index event, and had a diagnosis of at least one of obesity-associated comorbidities on or before the index event. Patients who were prescribed other GLP-1RAs or had bariatric surgery on or before the index event were excluded. The outcome recurrent CUD in patients with a prior history of CUD was defined as a medical encounter for CUD diagnosis after the index event. This study population was then divided into two cohorts: (1) Semaglutide cohort – 688 patients prescribed semaglutide, and (2) non-GLP1-RA anti-obesity medication cohort – 1,346 patients prescribed non-GLP-1RA anti-obesity medications but not semaglutide.

#### The study populations with T2D

The analyses on the associations of semaglutide with both incident and recurrent CUD among patients with T2D had a starting time of 12/2017 when semaglutide was approved in the US as Ozempic for treating T2D and an ending date of 5/2021 to allow us to separately examine the effects of semaglutide on CUD as Ozempic from those as Wegovy in the study population with obesity. Since patients in the study population with obesity were for the first time prescribed semaglutide after 6/2021, there was no overlap in the exposure cohorts for these two study populations.

To assess the association of semaglutide with incident CUD, the study population included 587,849 patients with T2D who had active medical encounters for T2D during 12/2017–5/2021, were for the first time prescribed semaglutide (Ozempic) or non-GLP1-1RA anti-diabetes medications (new-user design) during 12/2017–5/2021(index event), had no diagnosis of CUD on or before the index event and had a diagnosis of at least one of obesity-associated comorbidities (T2D, hypertension, hypercholesterolemia, hyperlipidemia, heart diseases, stroke) on or before the index event. The status of non-GLP1RA anti-diabetes medications was determined by the Anatomical Therapeutic Chemical or ATC code A10 “Drugs used in diabetes” with GLP1R agonizts (ATC code A10BJ “ Glucagon-like peptide-1 (GLP-1) analogs”) excluded. The list of non-GLP1RA anti-diabetes medications included insulins, metformin, sulfonylureas, alpha glucosidase inhibitors, thiazolidinediones, dipeptidyl peptidase 4 (DPP-4) inhibitors, sodium-glucose co-transporter 2 (SGLT2) inhibitors. Patients who were prescribed other GLP-1RAs or had bariatric surgery on or before the index event were excluded. This study population was divided into two cohorts: (1) Semaglutide cohort – 25,843 patients prescribed semaglutide, and (2) Non-GLP-1RA anti-diabetes medication cohort – 562,006 patients prescribed non-GLP-1RA anti-diabetes medications but not semaglutide.

To assess the associations of semaglutide with recurrent CUD, the study population included 8196 patients with T2D who had active medical encounters for T2D diagnosis in 12/2017–5/2021, were for the first time prescribed semaglutide or non-GLP1-1RA anti-diabetes medications during 12/2017–5/2021(index event), had a diagnosis of CUD on or before the index event, and had a diagnosis of at least one of obesity-associated comorbidities on or before the index event. Patients who were prescribed other GLP-1RAs or had bariatric surgery on or before the index event were excluded. This study population was then divided into two cohorts: (1) Semaglutide cohort – 254 patients prescribed semaglutide, and (2) non-GLP1-RA anti-diabetes medication cohort – 7942 patients prescribed non-GLP-1RA anti-diabetes medications but not semaglutide.

### Statistical analysis

For each study population, the Semaglutide cohort and the comparison cohort were propensity-score matched (1:1 using nearest neighbor greedy matching with a caliper of 0.25 times the standard deviation) on covariates that are potential risk factors for CUD [42, 43] including demographics, adverse socioeconomic determinants of health (e.g., problems related to education and literacy, employment and unemployment, housing and economic circumstances, social environment, upbringing, primary support group including family circumstances and various psychosocial circumstances), problems with lifestyle (e.g., tobacco use, lack of physical exercise, inappropriate diet and eating habits, high-risk sexual behavior, gambling and betting, and other problems related to lifestyle including antisocial behaviors and sleep deprivation), pre-existing medical conditions and medications. Obesity sub-categories were also matched to control obesity severity which included 3 ICD-10 diagnosis codes and 15 BMI categories ranging from BMI 30 to BMI 70 or greater (more details in Tables 1 and 2 and Supplementary Tables S1–3 in the Supplementary Appendix). The outcome –incident or recurrent diagnosis of CUD (International Classification of Diseases, Tenth Revision (ICD-10) code F12 “Cannabis related disorders”) – that occurred within the 12-month time window after the index event) were compared between matched cohorts. Recurrent CUD in patients with a prior history of CUD was defined as a medical encounter for CUD diagnosis (ICD-10 F12) after the index event. Kaplan–Meier analysis was used to estimate the probability of outcome at daily time intervals with censoring applied. When the last fact (the outcome of interests or other medical encounters) in the patient’s record is in the time window for analysis, the patient was censored on the day after the last fact in their record. Hazard ratio (HR) and 95% confidence intervals were used to describe the relative hazard of the outcomes based on a comparison of time to event rates.

**Table 1:** Characteristics of the Semaglutide cohort and the non-GLP-1RA anti-obesity medications cohort for the study population with obesity who had no prior history of CUD.

	Before propensity-score matching			After propensity-score matching		
	Semaglutide cohort	Non-GLP-1RA anti-obesity medications cohort	SMD	Semaglutide cohort	Non-GLP-1RA anti-obesity medications cohort	SMD
Total number	45,445	37,744		26,784	26,784	
Age at index event (years, mean ± SD)	53.2 ± 13.3	50.2 ± 15.1	0.21^a^	51.3 ± 13.2	51.2 ± 15.0	0.007
Sex (%)						
Female	61.9	64.7	0.06	65.8	65.3	0.01
Male	33.4	30.3	0.07	29.3	29.8	0.01
Unknown	4.7	5.0	0.01	5.0	4.9	0.002
Ethnicity (%)						
Hispanic/Latinx	7.4	6.3	0.04	6.6	6.7	0.007
Not Hispanic/Latinx	69.0	73.8	0.11^a^	71.1	71.3	0.004
Unknown	23.6	19.9	0.09	22.3	22.0	0.009
Race (%)						
Asian	2.4	1.0	0.11^a^	1.2	1.3	0.01
Black	16.4	16.1	0.007	16.0	16.1	0.003
White	64.7	66.9	0.05	66.7	66.3	0.009
Unknown	11.8	11.8	0.001	11.9	11.9	0.001
Marital status (%)						
Never Married	11.7	15.9	0.12^a^	13.2	13.5	0.006
Divorced	5.5	6.1	0.03	5.7	5.7	0.001
Widowed	3.0	3.3	0.02	3.0	3.0	0.001
Adverse socioeconomic determinants of health (%)	4.7	6.2	0.07	5.3	5.4	0.002
Problems related to lifestyle (%)	8.5	11.2	0.09	9.5	9.6	0.003
Obesity categories (%)						
Morbid (severe) obesity due to excess calories	59.8	49.1	0.22^a^	55.3	54.8	0.01
Obesity, unspecified	63.8	61.0	0.06	62.1	61.9	0.003
Other obesity due to excess calories	15.3	12.1	0.09	14.0	13.9	0.003
BMI 30.0–30.9	6.3	7.8	0.06	6.8	6.7	0.004
BMI 31.0–31.9	7.0	8.2	0.05	7.3	7.3	<.001
BMI 32.0–32.9	7.7	8.8	0.04	8.0	8.1	0.006
BMI 33.0–33.9	8.3	8.8	0.02	8.4	8.4	0.001
BMI 34.0–34.9	9.1	8.8	0.01	8.7	8.8	0.002
BMI 35.0–35.9	10.6	9.7	0.03	9.9	9.9	0.001
BMI 36.0–36.9	9.7	8.6	0.04	8.9	8.9	0.001
BMI 37.0–37.9	9.5	8.2	0.05	8.8	8.6	0.004
BMI 38.0–38.9	9.2	7.8	0.05	8.5	8.4	0.004
BMI 39.0–39.9	8.3	6.7	0.06	7.6	7.4	0.008
BMI 40.0–44.9	22.5	19.7	0.07	21.6	21.3	0.008
BMI 45.0–49.9	13.5	11.0	0.08	12.5	12.3	0.007
BMI 50.0–59.9	9.2	7.4	0.07	8.3	8.3	0.001
BMI 60.0–69.9	2.4	2.1	0.02	2.3	2.2	0.01
BMI ≥70	0.8	0.9	0.004	0.9	0.9	0.001
Family history of mental and behavioral disorders	0.7	1.1	0.04	0.8	0.8	0.003
Pre-existing medical conditions, procedures, medications (%)						
Type 2 diabetes	56.4	24.5	0.69^a^	33.0	33.4	0.008
Depression	29.7	39.9	0.22^a^	35.6	35.3	0.007
Mood disorders	34.9	48.7	0.28^a^	42.4	42.0	0.008
Anxiety disorders	40.6	50.0	0.19^a^	46.9	46.6	0.006
Psychotic disorders	1.3	2.3	0.08	1.7	1.7	<.001
Behavioral disorders	9.4	8.8	0.02	9.7	9.4	0.01
Disorders of adult personality and behavior	1.2	1.8	0.05	1.4	1.5	0.002
Behavioral and emotional disorders with onset usually occurring in childhood and adolescence	4.7	6.3	0.07	5.8	5.9	0.001
Conduct disorders	0.3	0.6	0.05	0.4	0.4	0.005
Symptoms and signs involving emotional state	5.2	6.8	0.07	5.9	5.8	0.006
Chronic pain	29.3	27.5	0.04	28.8	28.4	0.01
Cancer	36.8	28.7	0.17^a^	32.8	32.4	0.008
Alcohol use disorder	2.8	5.8	0.15^a^	3.6	3.6	0.002
Opioid use disorder	1.6	2.4	0.06	1.9	1.9	0.003
Tobacco use disorder	13.0	20.9	0.21^a^	15.9	16.0	0.003
Cocaine use disorder	0.4	0.9	0.07	0.5	0.6	0.01
Other stimulant disorders	0.4	0.9	0.06	0.5	0.5	0.001
Other psychoactive substance related disorders	1.1	2.1	0.08	1.4	1.4	0.004
Hypertension	75.3	70.8	0.10^a^	71.5	71.2	0.007
Disorders of lipoprotein metabolism and other lipidemias	73.7	61.5	0.26^a^	67.3	67.2	0.001
Hyperlipidemia	52.5	42.4	0.20^a^	46.3	46.1	0.003
Hypercholesterolemia	22.9	16.1	0.17^a^	18.9	18.7	0.007
Ischemic heart diseases	17.4	14.3	0.09	14.8	14.6	0.008
Other forms of heart disease	31.7	30.9	0.02	30.4	29.9	0.01
Cerebral infarction	3.1	3.4	0.02	3.2	3.1	0.005
Cerebrovascular diseases	7.2	7.8	0.02	7.2	7.1	0.006
Epilepsy and recurrent seizures	1.6	2.7	0.08	2.0	2.0	0.001
Migraine	12.9	16.3	0.09	15.5	15.3	0.004
Post-traumatic stress disorder (PTSD)	2.8	4.5	0.09	3.5	3.5	0.003
Substance abuse treatment	0.1	0.5	0.08	0.1	0.1	0.002
Psychotherapy	4.3	4.6	0.02	4.6	4.6	0.002
Zolpidem	8.9	7.4	0.06	8.4	8.2	0.008
Buspirone	5.4	7.4	0.08	6.6	6.5	0.002
Gabapentin	23.5	24.0	0.01	23.8	23.2	0.01

Shown were cohorts before and after propensity-score matching for the listed variables with their status based on the presence of related clinical codes anytime to the day of the index event. Shown were cohorts before and after propensity-score matching for the listed variables with their status based on the presence of related clinical codes anytime on or before the index event (the first prescription of semaglutide, or non-GLP-1RA anti-obesity medications during 6/2021–12/2022). Adverse socioeconomic determinants of health include problems related to education and literacy, employment and unemployment, housing and economic circumstances, social environment, upbringing, primary support group including family circumstances, various psychosocial circumstances. Problems with lifestyle included tobacco use, lack of physical exercise, inappropriate diet and eating habits, high-risk sexual behavior, gambling and betting, and other problems related to lifestyle including antisocial behavior and sleep deprivation.

*SMD* standardized mean differences, *SD* standard deviation.

^a^SMD greater than 0.1, a threshold indicating cohort imbalance.

**Table 2:** Characteristics of the Semaglutide cohort and the non-GLP-1RA anti-diabetes medications cohort for the study population with T2D who had no prior history of CUD.

	Before propensity-score matching			After propensity-score matching		
	Semaglutide cohort	Non-GLP-1RA anti-diabetes medications cohort	SMD	Semaglutide cohort	Non-GLP-1RA anti-diabetes medications cohort	SMD
Total number	25,843	562,006		25,820	25,820	
Age at index event (years, mean ± SD)	58.2 ± 11.9	63.1 ± 13.3	0.39^a^	58.2 ± 11.9	57.7 ± 13.4	0.04
Sex (%)						
Female	45.7	44.8	0.02	45.6	45.2	0.008
Male	47.2	51.5	0.09	47.2	47.5	0.005
Unknown	7.1	3.7	0.15^a^	7.1	7.3	0.006
Ethnicity (%)						
Hispanic/Latinx	6.7	8.8	0.08	6.7	6.6	0.004
Not Hispanic/Latinx	64.1	56.9	0.15^a^	64.1	65.4	0.03
Unknown	29.3	34.2	0.11^a^	29.3	28.1	0.03
Race (%)						
Asian	4.8	5.4	0.03	4.7	4.8	0.002
Black	14.9	17.4	0.07	14.9	14.6	0.01
White	60.9	58.8	0.04	60.9	61.5	0.01
Unknown	14.3	13.5	0.02	14.3	14.3	0.001
Marital status (%)						
Never Married	10.3	9.7	0.02	10.3	10.3	0.001
Divorced	5.3	4.3	0.05	5.3	5.3	0.002
Widowed	4.8	5.9	0.05	4.8	4.6	0.009
Adverse socioeconomic determinants of health (%)	3.0	2.2	0.05	3.0	2.7	0.02
Problems related to lifestyle (%)	6.9	4.3	0.11^a^	6.9	6.7	0.009
Family history of mental and behavioral disorders	0.4	0.3	0.01	0.4	0.4	0.009
Pre-existing medical conditions, procedures, medications (%)						
Depression	21.8	13.4	0.22^a^	21.7	20.7	0.03
Mood disorders	25.2	16.4	0.22^a^	25.2	24.2	0.02
Anxiety disorders	26.3	16.0	0.25^a^	26.2	25.5	0.02
Psychotic disorders	1.3	2.0	0.05	1.3	1.2	0.02
Behavioral disorders	5.5	1.8	0.20^a^	5.5	5.2	0.01
Disorders of adult personality and behavior	0.9	0.5	0.05	0.9	0.8	0.008
Behavioral and emotional disorders with onset usually occurring in childhood and adolescence	2.0	0.8	0.10^a^	2.0	2.0	0.004
Conduct disorders	0.2	0.1	0.009	0.2	0.2	0.007
Symptoms and signs involving emotional state	3.5	2.2	0.08	3.5	3.2	0.02
Chronic pain	21.0	10.6	0.29^a^	20.9	20.2	0.02
Cancer	32.5	18.9	0.32^a^	32.5	32.0	0.01
Alcohol use disorder	2.3	3.3	0.06	2.3	2.0	0.02
Opioid use disorder	1.2	1.0	0.02	1.2	1.1	0.01
Tobacco use disorder	13.7	13.9	0.007	13.7	13.2	0.01
Cocaine use disorder	0.4	0.7	0.03	0.4	0.3	0.02
Other stimulant disorders	0.3	0.4	0.02	0.3	0.3	<.001
Other psychoactive substance related disorders	0.9	0.8	0.01	0.9	0.9	0.005
Hypertension	86.1	87.1	0.03	86.1	85.3	0.02
Disorders of lipoprotein metabolism and other lipidemias	85.9	65.5	0.49^a^	85.9	86.9	0.03
Hyperlipidemia	64.8	49.9	0.31^a^	64.8	64.4	0.007
Hypercholesterolemia	27.1	16.6	0.26^a^	27.1	26.6	0.01
Ischemic heart diseases	24.2	27.0	0.06	24.2	22.8	0.03
Other forms of heart disease	30.7	34.0	0.07	30.7	28.6	0.05
Cerebral infarction	4.7	5.9	0.06	4.7	4.2	0.02
Cerebrovascular diseases	10.2	11.9	0.05	10.2	9.1	0.04
Epilepsy and recurrent seizures	1.5	2.1	0.05	1.5	1.3	0.01
Migraine	6.7	2.8	0.19^a^	6.7	6.4	0.01
Post-traumatic stress disorder (PTSD)	1.6	1.1	0.04	1.6	1.4	0.01
Morbid (severe) obesity due to excess calories	28.0	11.9	0.41^a^	28.0	27.0	0.02
Obesity, unspecified	40.0	18.7	0.48^a^	39.9	39.0	0.02
Other obesity due to excess calories	7.7	1.9	0.27^a^	7.7	6.9	0.03
BMI 30.0–30.9	4.8	2.1	0.15^a^	4.8	4.6	0.01
BMI 31.0–31.9	5.4	2.1	0.17^a^	5.4	5.0	0.02
BMI 32.0–32.9	5.7	2.2	0.18^a^	5.7	5.4	0.01
BMI 33.0–33.9	5.8	2.1	0.19^a^	5.8	5.4	0.02
BMI 34.0–34.9	6.0	2.0	0.20^a^	6.0	5.5	0.02
BMI 35.0–35.9	6.6	2.2	0.22^a^	6.5	6.1	0.02
BMI 36.0–36.9	5.7	1.9	0.20^a^	5.7	5.2	0.02
BMI 37.0–37.9	5.4	1.7	0.20^a^	5.4	5.0	0.02
BMI 38.0–38.9	4.9	1.6	0.19^a^	4.9	4.7	0.01
BMI 39.0–39.9	3.8	1.3	0.16^a^	3.8	3.6	0.01
BMI 40.0–44.9	11.4	4.7	0.25^a^	11.3	10.9	0.01
BMI 45.0–49.9	6.2	2.4	0.18^a^	6.2	5.7	0.02
BMI 50.0–59.9	4.0	1.7	0.14^a^	4.0	3.7	0.02
BMI 60.0–69.9	1.0	0.4	0.07	1.0	1.0	<.001
BMI ≥70	0.4	0.2	0.04	0.4	0.4	0.001
Substance abuse treatment	0.1	0.2	0.03	0.1	0.1	0.01
Psychotherapy	2.3	0.6	0.14^a^	2.3	2.1	0.009
Zolpidem	7.6	3.3	0.19^a^	7.6	7.0	0.02
Buspirone	2.8	1.4	0.09	2.8	2.5	0.01
Gabapentin	23.3	14.2	0.23^a^	23.3	22.3	0.02

Shown were cohorts before and after propensity-score matching for the listed variables with their status based on the presence of related clinical codes anytime to the day of the index event. Shown were cohorts before and after propensity-score matching for the listed variables with their status based on the presence of related clinical codes anytime on or before the index event (the first prescription of semaglutide, or non-GLP-1RA anti-diabetes medications during 12/2017–5/2021). Adverse socioeconomic determinants of health include problems related to education and literacy, employment and unemployment, housing and economic circumstances, social environment, upbringing, primary support group including family circumstances, and various psychosocial circumstances. Problems with lifestyle included tobacco use, lack of physical exercise, inappropriate diet and eating habits, high-risk sexual behavior, gambling and betting, and other problems related to lifestyle including antisocial behavior and sleep deprivation.

*SMD* standardized mean differences, *SD* standard deviation.

^a^SMD greater than 0.1, a threshold indicating cohort imbalance.

Separate analyses were performed in patients stratified by sex (women, men), age groups (≤55, >55 years), and race (Black, White). For the study population with obesity, a separate analysis was performed in patients with T2D and patients without T2D. For the study population with T2D, a separate analysis was performed in patients with obesity and patients without obesity.

To examine longer-term associations of semaglutide with CUD, the outcome –incident and recurrent diagnosis of CUD– in patients with T2D was followed for 1-, 2-, and 3-year following the index event (the first prescription of semaglutide vs. non-GLP-1 RA anti-diabetes medications occurred during 12/2017-5/2021). Similar analyses were not performed for patients with obesity since semaglutide was approved as Wegovey for weight management in 6/2021.

The data were collected and analyzed on January 21, 2024, within the TriNetX Analytics Platform. Details of clinical codes for eligibility criteria, exposure, outcomes, and confounders are in Supplementary Table S1 in the Supplementary Appendix.

## Results

### Associations of semaglutide with incident and recurrent CUD diagnosis in patients with obesity

The study population for the analyses of incident CUD diagnosis in patients with obesity consisted of 83,189 patients with obesity who had no prior recorded diagnosis of CUD. The Semaglutide cohort compared with the Non-GLP-1RA anti-obesity medications cohort was older and had a higher prevalence of morbid obesity, T2D and obesity-associated comorbidities, and a lower prevalence of mental disorders and substance use disorders. After propensity-score matching, the two cohorts (26,784 in each cohort, mean age 51.3 years, 65.6% women, 16.1% Black, 66.5% White, 6.7% Hispanic) were balanced (Table 1).

The study population for the analysis of recurrent CUD diagnosis or medical encounter for CUD diagnosis in patients with obesity who had a prior diagnosis of CUD consisted of 2,034 patients. The Semaglutide cohort compared to the non-GLP-1RA anti-obesity medications cohort was older, had a lower prevalence of adverse socioeconomic determinants of health, mental disorders, and substance use disorders, and a higher prevalence of morbid obesity, T2D and obesity-associated comorbidities. After propensity-score matching, the two cohorts (504 in each cohort, mean age 46.1 years, 57.5% women, 26.4% black, 54.7% white, 6.7% Hispanic) were balanced (see Supplementary Table S2 in the Supplementary Appendix).

**Fig. 1:**
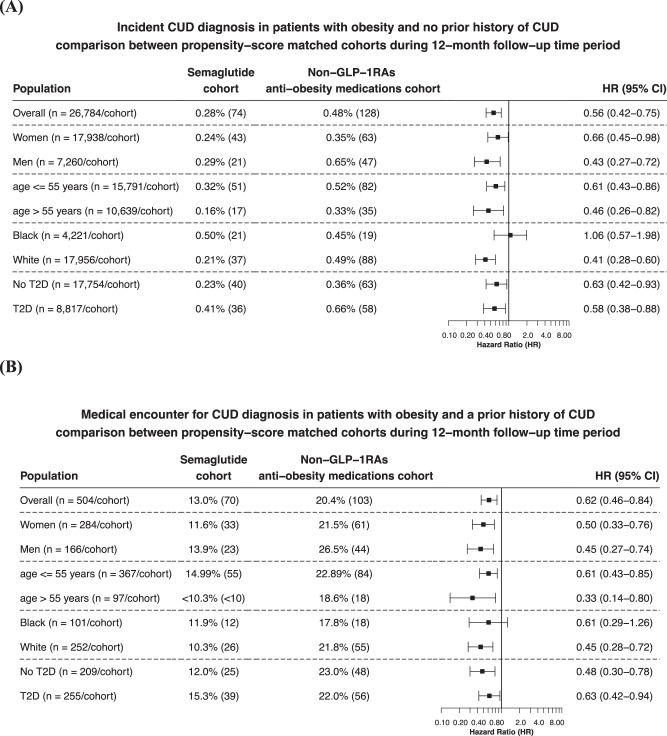
Incident and recurrent CUD diagnosis in patients with obesity. **A** Comparison of incident CUD diagnosis (new diagnosis) in patients with obesity who had no prior history of CUD between propensity-score matched Semaglutide and Non-GLP-1RA anti-obesity medications cohorts, stratified by gender, age group, race and the status of type 2 diabetes. **B** Comparison of medical encounters for CUD diagnosis (recurrent CUD diagnosis) in patients with obesity who had a prior history of CUD between propensity-score matched Semaglutide and Non-GLP-1RA anti-obesity medications cohorts, stratified by gender, age group, race and the status of type 2 diabetes. Outcomes were followed for 12 months following the index event (first prescription of semaglutide or non-GLP-1 RA anti-obesity medications during 6/2021–12/2022). Hazard rates were calculated using Kaplan–Meier analysis to estimate the probability of outcome at daily time intervals with censoring applied. Overall risk = number of patients with outcomes during the 12-month time window/number of patients in the cohort at the beginning of the time window.

Among patients with no prior history of CUD, semaglutide was associated with a significantly lower risk for incident CUD diagnosis compared with non-GLP-1RA anti-obesity medications for a 12-month follow-up period (0.28% vs. 0.48%; HR: 0.56, 95% CI: 0.42–0.75). Consistent reductions with semaglutide were seen in patients stratified by gender, age group, and race, except in Black patients, with the biggest reduction in men and patients aged >55 years old. A similar lower risk for incident CUD was observed in patients with and without T2D (Fig. 1A).

Among patients with a prior history of CUD, semaglutide was associated with a significantly lower risk for recurrent CUD diagnosis (medical encounters for CUD diagnosis) compared with non-GLP-1RA anti-obesity medications for a 12-month follow-up period (13.0% vs. 20.4%; HR: 0.62, 95% CI: 0.46–0.84). Consistent reductions with semaglutide were seen in patients stratified by gender, age group, and race, except in Black patients, with the biggest reduction in men and patients aged >55 years old. A similar lower risk for recurrent CUD was observed in patients with and without T2D (Fig. 1B).

### Associations of semaglutide with incident and recurrent CUD diagnosis in patients with type 2 diabetes

The study population for the analyses of incident CUD diagnosis in patients with T2D consisted of 587,849 patients with T2D who had no prior recorded diagnosis of CUD. The Semaglutide cohort compared with the Non-GLP-1RA anti-diabetes medications cohort was younger and had a higher prevalence of obesity, obesity-associated comorbidities and mental disorders. After propensity-score matching, the two cohorts (25,820 in each cohort, mean age 58.0 years, 45.4% women, 14.8% Black, 61.2% White, 6.7% Hispanic) were balanced (Table 2).

The study population for the analysis of recurrent CUD diagnosis in patients with T2D who had a prior diagnosis of CUD consisted of 8,196 patients. The Semaglutide cohort compared to the non-GLP-1RA anti-diabetes medications cohort included more women, had a higher prevalence of problems with lifestyle, obesity, obesity-associated comorbidities, mental disorders, and substance use disorders. After propensity-score matching, the two cohorts (241 in each cohort, mean age 51.8 years, 39.0% women, 32.6% black, 44.4% white, 5.8% Hispanic) were balanced (see Supplementary Table S3 in the Supplementary Appendix).

**Fig. 2:**
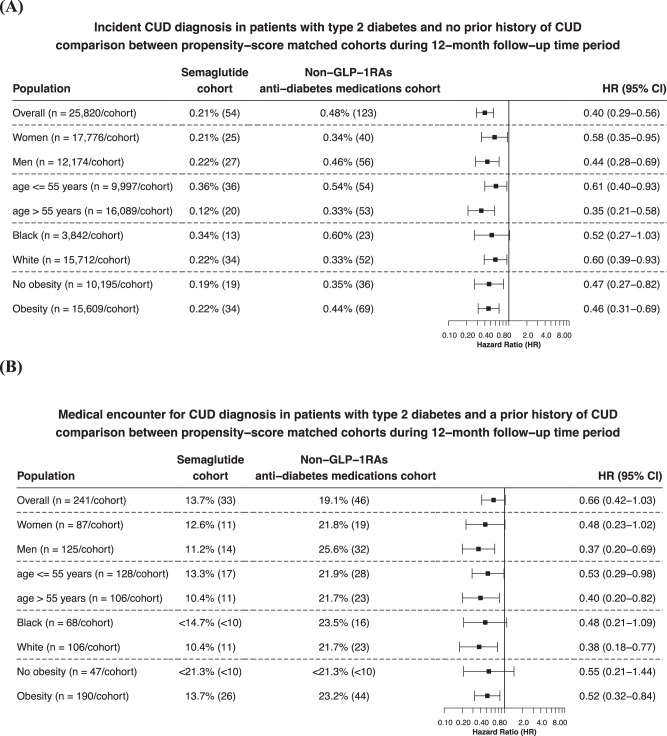
Incident and recurrent CUD diagnosis in patients with type 2 diabetes. **A** Comparison of incident CUD diagnosis (new diagnosis) in patients with type 2 diabetes who had no prior history of CUD between propensity-score matched Semaglutide and Non-GLP-1RA anti-diabetes medications cohorts, stratified by gender, age group, race and the status of obesity. **B** Comparison of medical encounters for CUD diagnosis in patients with type 2 diabetes who had a prior history of CUD between propensity-score matched Semaglutide and Non-GLP-1RA anti-diabetes medications cohorts, stratified by gender, age group, race and the status of obesity. Outcomes were followed for 12 months following the index event (first prescription of semaglutide or non-GLP-1 RA anti-diabetes medications during 12/2017–5/2021). Hazard rates were calculated using Kaplan–Meier analysis to estimate the probability of outcome at daily time intervals with censoring applied. Overall risk = number of patients with outcomes during the 12-month time window/number of patients in the cohort at the beginning of the time window.

Among patients with T2D who had no prior history of CUD, semaglutide was associated with a significantly lower risk for incident CUD diagnosis compared with non-GLP-1RA anti-diabetes medications for a 12-month follow-up period (0.21% vs. 0.48%; HR: 0.40, 95% CI: 0.29–0.56). Consistent reductions with semaglutide were seen in patients stratified by gender, age group, and race, with the biggest reduction in men, white, and patients aged >55 years old. A similar lower risk for incident CUD was observed in patients with and without obesity (Fig. 2A).

Among patients with T2D who had a prior history of CUD, semaglutide was associated with a lower risk for recurrent CUD diagnosis (medical encounters for CUD diagnosis) compared with non-GLP-1RA anti-diabetes medications for a 12-month follow-up period (13.7% vs. 19.1%; HR: 0.66, 95% CI: 0.42–1.03). Consistent reductions with semaglutide were seen in patients stratified by gender, age group, and race. A similar lower risk for recurrent CUD was observed in patients with and without obesity, though not statistically significant in patients without obesity (Fig. 2B).

**Fig. 3:**
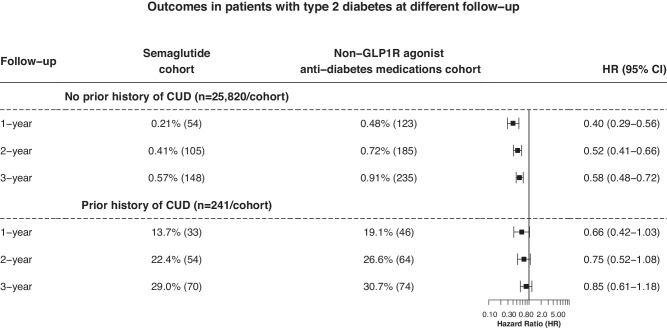
Comparison of incident and recurrent CUD in patients with T2D at longer follow-up between propensity-score matched Semaglutide and non-GLP-1RA anti-diabetes medications cohorts. Hazard rates were calculated using Kaplan–Meier analysis to estimate the probability of outcome at daily time intervals with censoring applied. Overall risk = number of patients with outcomes during the follow-up time window/number of patients in the cohort at the beginning of the time window.

We then examined the longer-term association of semaglutide with both incident and recurrent diagnosis of CUD in patients with T2D. Compared with non-GLP-1RA anti-diabetes medications, semaglutide was associated with a significantly lower risk of incident CUD, though the association attenuated over time. Among patients with a prior history of CUD, semaglutide was associated with a lower, but not statistically significant, risk of recurrent CUD and the strength of the association attenuated over time (Fig. 3).

## Discussion

Here we document a potential beneficial effect of semaglutide on both the incidence and recurrence of CUD in two separate real-world populations, one with obesity and the other with T2D. These results are consistent with reports that patients treated with semaglutide notice reduced interest in consuming addictive substances including alcohol or tobacco [12]. Currently, several registered clinical trials are ongoing to evaluate the effect of semaglutide on alcohol consumption [13–17] and on smoking cessation [18, 19]. However, little attention has been placed on the effects of semaglutide or other GLP1-RAs on cannabis consumption even though the prevalence of cannabis use is even higher than that of tobacco use among certain demographics. While preclinical studies have investigated the effects of GLP-1RAs on nicotine, alcohol, cocaine, or opioids [20–23], to our knowledge no studies have been reported on their effects of tetrahydrocannabinol (THC), the major psychoactive component in cannabis. The potential beneficial effects of semaglutide (and presumably other GLP1R agonizts) in the treatment of CUD deserve attention in that currently there are no approved medications for its treatment [44]. As such the only available proven effective therapeutic alternatives are psychosocial interventions, but their effects tend to be limited [45]. Thus, our results support conducting preclinical studies of semaglutide (and other GLP1R agonist) on THC and randomized clinical trials to evaluate the therapeutic benefits of semaglutide in CUD in individuals with obesity or T2D as well as in patients with CUD who don’t have these co-morbid conditions.

Though the mechanism(s) underlying the decreased risk of incident and recurrent CUD with semaglutide is unclear, several mechanisms could be involved. Since our results document not just a reduction in relapse but also in the incidence of CUD, one could question the mechanism by which semaglutide prevents the transition from cannabis use to CUD. Though the majority of cannabis users do not proceed to develop a CUD, approximately 10% do, and among young users, the risk can increase to 30% [46]. This transition is typically associated with an increase in the regularity and frequency of use [47]. In the case of nicotine, preclinical studies showed evidence that the rewarding effects of nicotine are reduced by stimulation of GLP-1 receptors in the medial habenula, which is a brain region that mediates nicotine’s aversive effects [48]; whereas exenatide a GLP-1R agonist drug attenuated nicotine’s activation of dopamine reward circuitry [49]. Whether similar mechanisms pertain to cannabis is unclear since there are no studies that have evaluated the effects of GLP1 signaling in the aversive or rewarding effects of THC. Cannabinoid receptor 1 (CB1R) is expressed in presynaptic and postsynaptic neuronal sites in the lateral habenula, where they modulate behavioral responses to stress exposure, such that CB1R blockade reduced anxiety-like behaviors [50] and also in GABAergic cells in the ventral tegmental area (VTA) and their stimulation increases firing of dopamine neurons, which modulate reward [51]. Indeed endocannabinoid signaling through CB1R regulates the activity of dopamine (DA) neurons in the VTA [52], which projects to the nucleus accumbens, a key brain reward region activated by addictive drugs including THC [53, 54]. CB1R signaling also inhibits the excitatory stimulation from the infralimbic cortex in response to stressful stimuli relayed via the bed nucleus of the stria terminalis (BNST) [55]. Since the BNST also expresses GLP1R and their ablation reduces anxiety-like behaviors [56], this could provide another potential convergence mechanism by which semaglutide or other GLP1R could affect cannabis consumption. Though to our knowledge there are no studies on interactions between GLP-1 and CB1 receptors in the habenula, VTA or other brain regions, there is evidence of reciprocal functional interactions between these two receptors peripherally [57].

In juxtaposition, the high prevalence of cannabis use alongside the rise in prescriptions of semaglutide for diabetes and obesity has led to concerns among cannabis users that their cannabis use could interfere with semaglutide therapeutic effects. Even though there is no evidence of interactions between semaglutide and cannabis nor of reports of reduced efficacy, this is an area that requires monitoring. In particular, since CB1Rs, the main target for the rewarding effects of cannabis, are expressed on incretin-secreting cells in rodents and may play a role in regulating GLP-1 secretion [58, 59]. In fact, CB1R antagonist drugs increased GLP-1 release and improved the response to GLP-1RA in rats with diet-induced obesity [58, 60]. Since THC is a partial CB1R agonist, a concern could be that it might inhibit GLP-1 release and attenuate the effects of semaglutide. On the other hand, and seemingly paradoxically in rats the CB1R agonist WIN 55,212-2 enhanced the anorexigenic effects of subthreshold doses of the GLP1-RA exendin-4 resulting in a significant decrease in food intake and body weight in rats [61]. Similar effects for an agonist and antagonist on food intake could reflect the dose dependency of cannabinoids effects on food intake, such that decreased intake is reported after high doses whereas low doses increase food consumption [61].

In interpreting the findings of our study, it is important to consider them within its limitations: First, this is a retrospective observational study, so no causal inferences can be drawn. Second, patients in our study represented those who had medical encounters with healthcare systems contributing to the TriNetX Platform. Though both the exposure and comparison cohorts were drawn from the same platform, which should not significantly impact the relative hazard rate ratios, results from the TriNetX platform need to be validated in other populations and platforms of EHRs. Third, retrospective observational studies have inherent limitations, including unmeasured or uncontrolled confounders and biases. Though we controlled for an extensive list of variables and the findings were replicated in two separate populations with different characteristics at two non-overlapping study periods, these limitations could not be fully eliminated. Future controlled trials are necessary to assess the associations of semaglutide with CUD. Fourth, in our study the follow-up time for the main analyses was 12 months. Though we conducted a longer-term follow-up analysis in the T2D population, future studies are necessary to evaluate long-term associations of semaglutide in patients with obesity and patients with T2D. Fifth, the higher dose format of semaglutide as Wegovy was approved for weight management, and the lower dose format of semaglutide as Ozempic was approved for treating T2D. Since Wegovy and Ozempic were approved for different indications, we could not directly assess the dosage effect of semaglutide on CUD in the same study population. Sixth, we could not directly control for patient adherence to medications due to limited medical adherence information captured in patient EHRs. Our study population included patients who had recent medical encounters for the diagnosis of obesity or T2D and were subsequently prescribed semaglutide or other anti-obesity or anti-diabetes medications, suggesting that patients had active obesity or T2D that needed medical attention and treatments. However, patients may discontinue medications for reasons such as financial burden, drug side effects, or lack of efficacy.

In summary, our results show that semaglutide was associated with a lower risk for both incident and relapse of CUD compared to non-GLP-1 RA anti-obesity and anti-diabetes medications. While these findings provide preliminary evidence of the potential benefit of semaglutide in CUD in real-world populations further preclinical studies are warranted to understand the underlying mechanism and randomized clinical trials are needed to support its use clinically for CUD.

## Supplementary information

Supplementary Appendix

## Data Availability

This study used population-level aggregate and de-identified data collected by the TriNetX Platform and are available from TriNetX, LLC (https://trinetx.com/) but third-party restrictions apply to the availability of these data. The data were used under license for this study with restrictions that do not allow for the data to be redistributed or made publicly available. To gain access to the data, a request can be made to TriNetX (join@trinetx.com), but costs might be incurred, and a data-sharing agreement would be necessary. Data specific to this study including diagnosis codes and cohort characteristics in aggregated format are included in the manuscript as tables, figures, and supplementary files.

## Code availability

All the statistical analyses in this study including propensity-score matching and Kaplan–Meier Survival analyses were conducted within the TriNetX platform by using its built-in functions. Data and code to reproduce the analyses can be accessed at https://github.com/bill-pipi/semaglutide_cannabis.
