# Supplementary material for: Association of semaglutide with reduced incidence and relapse of cannabis use disorder in real-world populations: a retrospective cohort study
Source: Mol Psychiatry. 2024 Mar 14;29(8):2587–98. doi: 10.1038/s41380-024-02498-5 (PMC11412894; doi:10.1038/s41380-024-02498-5)
Supplement: Supplementary file 1 — Supplementary Appendix [file 41380_2024_2498_MOESM1_ESM.docx]

**Supplementary Appendix**

Table of Contents

[List of Investigators 1](#_Toc148961287)

[Database 1](#_Toc148961288)

[Supplementary Tables 2](#_Toc148961289)

# **List of investigators**

William Wang^1^, Nora D. Volkow^2*^, Nathan A. Berger^1^, Pamela B Davis^3^, David C. Kaelber^4^, Rong Xu^5^

^1^Center for Science, Health, and Society, Case Western Reserve University School of Medicine, Cleveland, OH, USA

^2^National Institute on Drug Abuse, National Institutes of Health, Bethesda, MD, USA

^3^Center for Community Health Integration, Case Western Reserve University School of Medicine, Cleveland, OH, USA

^4^Center for Clinical Informatics Research and Education, The MetroHealth System, Cleveland, OH, USA

^5^Center for Artificial Intelligence in Drug Discovery, Case Western Reserve University School of Medicine, Cleveland, OH, USA

# **Database**

The data used in this study were collected and analyzed on January 21, 2024 within the TriNetX Analytics platform based on the “Research US Collaborative Network”. We used the TriNetX platform to access aggregated and de-identified electronic health records (EHRs) of 105.3 million patients from 61 healthcare organizations in the US across 50 states, covering diverse geographic regions, age, race/ethnic, income and insurance groups and clinical setting. TriNetX, LLC is compliant with the Health Insurance Portability and Accountability Act (HIPAA). Any data displayed on the TriNetX Platform in aggregate form, or any patient level data provided in a data set generated by the TriNetX Platform only contains de-identified data as per the de-identification standard defined in Section §164.514(a) of the HIPAA Privacy Rule. TriNetX built-in analytic functions (e.g., incidence, prevalence, outcomes analysis, survival analysis, propensity score matching) allow for patient-level analyses, while only reporting population level data. The MetroHealth System, Cleveland OH, IRB determined research using TriNetX, in the way described here, is not Human Subject Research and therefore IRB exempt.

TriNetX is a platform that de-identifies and aggregates electronic health record (EHR) data from contributing healthcare systems, most of which are large academic medical institutions with both inpatient and outpatient facilities at multiple locations, across all 50 states in the US. TriNetX Analytics provides web-based and secure access to patient EHR data from hospitals, primary care, and specialty treatment providers, covering diverse geographic locations, age groups, racial and ethnic groups, income levels and insurance types including various commercial insurances, governmental insurance (Medicare and Medicaid), self-pay/uninsured, worker compensation insurance, military/VA insurance among others.

Self-reported sex (female, male), race and ethnicity data in TriNetX comes from the underlying clinical EHR systems of the contributing healthcare systems. TriNetX maps race and ethnicity data from the contributing healthcare systems to the following categories: (1) Race: Asian, American Indian or Alaskan Native, Black or African American, Native Hawaiian or Other, White, Unknown race; and (2) Ethnicity: Hispanic or Latino, Not Hispanic or Latino, Unknown Ethnicity.

TriNetX completes an intensive data preprocessing stage to minimize missing values. TriNetX maps the data to a consistent clinical data model with a consistent semantic meaning so that the data can be queried consistently regardless of the underlying data source. All covariates are either binary, categorical (which expands to a set of binary columns), or continuous but essentially guaranteed to exist. Age is guaranteed to exist. Missing sex values are represented using “Unknown Sex”. The missing data for race and ethnicity are presented as “Unknown race” or “Unknown Ethnicity”. For other variables including medical conditions, procedures, lab tests, and socio-economic determinant health, the value is either present or absent so “missing” is not pertinent.

# **Supplementary Tables**

**Table S1**: Clinical diagnosis, and other codes used in TriNetX platform that are used to determine the status of variables for study population definitions, exposures, outcomes, and for propensity-score matching for cohorts.

| **Eligibility**  **(inclusion and exclusion criteria)** | CUD | - Cannabis related disorders (ICD-10 code: F12) | present/absent |
| --- | --- | --- | --- |
|  | Obesity | - Obesity due to excess calories (ICD-10 code: E66.0) - Morbid (severe) obesity due to excess calories (ICD-10 code: E66.01) - Drug-induced obesity (ICD-10 code: E66.1) - Morbid (severe) obesity with alveolar hypoventilation (ICD-10 code: E66.2) - Other obesity (ICD-10 code: E66.8) - Obesity, unspecified (ICD-10 code: E66.9) - Body mass index [BMI] 30-39, adult (ICD-10 code: Z68.3) - Body mass index [BMI] 40 or greater, adult (ICD-10 code: Z68.4) - Body mass index [BMI] 30.0-30.9, adult (ICD-10 code: Z68.30) - Body mass index [BMI] 31.0-31.9, adult (ICD-10 code: Z68.31) - Body mass index [BMI] 32.0-32.9, adult   (ICD-10 code: Z68.32)   - Body mass index [BMI] 33.0-33.9, adult (ICD-10 code: Z68.33) - Body mass index [BMI] 34.0-34.9, adult   (ICD-10 code: Z68.34)   - Body mass index [BMI] 35.0-35.9, adult   (ICD-10 code: Z68.35)   - Body mass index [BMI] 36.0-36.9, adult (ICD-10 code: Z68.36) - Body mass index [BMI] 37.0-37.9, adult (ICD-10 code: Z68.37) - Body mass index [BMI] 38.0-38.9, adult (ICD-10 code: Z68.38) - Body mass index [BMI] 39.0-39.9, adult (ICD-10 code: Z68.39) - Body mass index [BMI] 40.0-44.9, adult (ICD-10 code: Z68.41) - Body mass index [BMI] 45.0-49.9, adult (ICD-10 code: Z68.42) - Body mass index [BMI] 50.0-59.9, adult (ICD-10 code: Z68.43) - Body mass index [BMI] 60.0-69.9, adult (ICD-10 code: Z68.44) - Body mass index [BMI] 70 or greater, adult (ICD-10 code: Z68.45) | present/absent |
|  | T2D | Type 2 diabetes mellitus (ICD-10 code: E11) | present/absent |
|  | Obesity-related comborbidities | - Type 2 diabetes mellitus (ICD-10 code: E11) - Hypertensive diseases (ICD-10 code: E11) - Disorders of lipoprotein metabolism and other lipidemias (ICD-10 code: E78) - Pure hypercholesterolemia (ICD-10 code: E78.0) - Mixed hyperlipidemia (ICD-10 code: E78.2) - Other hyperlipidemia (ICD-10 code: E78.2) - Hyperlipidemia, unspecified (ICD-10 code: E78.5) - Ischemic heart diseases (ICD-10 code: I20-I25) - Other forms of heart disease (ICD-10 code: I30-I5A) - Cerebral infarction (ICD-10 code: I63)   Cerebrovascular diseases (ICD-10 code: I60-I69) | present/absent |
|  | Bariatric surgery | - Bariatric surgery status (ICD-10 code: Z98.84) - Gastrointestinal System / Bypass / Stomach (ICD10 code: :0D16 | present/absent |
|  | Other GLP-1RA medications | - lixisenatide (RxNorm code: 1440051) - albiglutide (RxNorm code: 1534763) - dulaglutide (RxNorm code: 1551291) - liraglutide (RxNorm code: 475968) - exenatide (RxNorm code: 60548) - tirzepatide (RxNorm code: 2601723) | present/absent |
| **Treatment strategies** | Semaglutide | Semaglutide (RxNorm code: 1991302) | present/absent |
|  | non-GLP-1RA anti-obesity medications | - Orlistat (RxNorm code: 37925) - Qsymia (Phentermine: RxNorm code 8152, Topiramate: RxNorm code 38404) - Contrave (bupropion: RxNorm code 42347, naltrexone: RxNorm code 7243) - Setmelanotide (RxNorm code: 2469247) | present/absent |
|  | Non- GLP-1RA anti-diabetic medications | - Drugs used in diabetes (ATC code: A10) with GLP-1RA medications (lixisenatide, albiglutide, dulaglutide, liraglutide, exenatide, tirzepatide) excluded. | present/absent |
| **Outcome** | CUD diagnosis | Cannabis related disorders (ICD-10 code: F12) | present/absent |
| **Demographics**  **(variables to be propensity-score matched)** | Age at the index event | Age | continuous |
|  | Female | F | present/absent |
|  | Male | M | present/absent |
|  | Asian | Asian (Demographics: 2028-9) | present/absent |
|  | Black or African American | Black or African American (Demographics: 2054-5) | present/absent |
|  | White | White (Demographics: 2106-3) | present/absent |
|  | Hispanic/Latino | Hispanic or Latino (Demographics: 2135-2) | present/absent |
|  | Not Hispanic or Latino | Not Hispanic or Latino (Demographics: 2186-5) | present/absent |
|  | Unknown race | Unknown Race (Demographics: 2131-1) | present/absent |
|  | Unknown ethnicity | Unknown Ethnicity (Demographics: UN) | present/absent |
|  | Divorced | Divorced (Demographics: D) | present/absent |
|  | Widowed | Widowed (Demographics: W) | present/absent |
|  | Never married | Never Married (Demographics: S) | present/absent |
| **Adverse socioeconomic determinants of health, lifestyle factors, medical conditions, medication prescriptions, (anytime to before or on the day of the index event)** | Adverse socioeconomic and psychosocial circumstances | Persons with potential health hazards related to socioeconomic and psychosocial circumstances (ICD-10 code: Z55-Z65)   - Problems related to education and literacy (ICD-10 code: Z55) - Problems related to employment and unemployment (ICD-10 code: Z56) - Problems related to housing and economic circumstances (ICD-10 code: Z59) - Problems related to social environment (ICD-10 code: Z60) - Problems related to upbringing (ICD-10 code: Z62) - Other problems related to primary support group, including family circumstances (ICD-10 code: Z63) - Problems related to certain psychosocial circumstances ICD-10 code: Z64) - Problems related to other psychosocial circumstances (ICD-10 code: Z65) | present/absent |
|  | Problems related to lifestyle | Problems related to lifestyle (ICD-10 code: Z72)   - Tobacco use (ICD-10 code: Z72.0) - Lack of physical exercise ((ICD-10 code: Z72.3) - Inappropriate diet and eating habits (ICD-10 code: Z72.4) - High risk sexual behavior (ICD-10 code: Z72.5) - Gambling and betting (ICD-10 code: Z72.6) - Other problems related to lifestyle (ICD-10 code: Z72.8)   Problem related to lifestyle, unspecified (ICD-10 code: Z72.9) | present/absent |
|  | Depression | Depressive episode (ICD-10 code: F32) | present/absent |
|  | Mood disorders | Mood [affective] disorders (ICD-10 code: F30-F39) | present/absent |
|  | Anxiety, dissociative, , somatoform and other nonpsychotic mental disorders including PTSD | Anxiety, dissociative, stress-related, somatoform and other nonpsychotic mental disorders (ICD-10 code: F40-F48) | present/absent |
|  | Schizophrenia, schizotypal, delusional, and other non-mood psychotic disorders | Schizophrenia, schizotypal, delusional, and other non-mood psychotic disorders (ICD-10 code: F20-F29) | present/absent |
|  | Behavioral disorders including sleep disorders | Behavioral syndromes associated with physiological disturbances and physical factors (ICD-10 code: F50-F59) | present/absent |
|  | Disorders of adult personality and behavior including impulse and gender identity disorders | Disorders of adult personality and behavior (ICD-10 code: F60-F69) | present/absent |
|  | Behavioral and emotional disorders with onset usually occurring in childhood and adolescence | Behavioral and emotional disorders with onset usually occurring in childhood and adolescence (ICD-10 code: F90-F98) | present/absent |
|  | Conduct disorders | Conduct disorders ((ICD-10 code: F90) | present/absent |
|  | Symptoms and signs involving emotional state | Symptoms and signs involving emotional state (ICD-10 code: R45) | present/absent |
|  | Chronic pain | Chronic pain, not elsewhere classified (ICD-10 code: G89.2) | present/absent |
|  | Alcohol use disorder | Alcohol use disorders (ICD-10 code: F10) | present/absent |
|  | Opioid use disorder | Opioid use disorders (ICD-10 code: F11) | present/absent |
|  | Tobacco use disorder | Nicotine dependence (ICD-10 code: F17) | present/absent |
|  | Cocaine use disorder | Cocaine use disorders (ICD-10 code: F14) | present/absent |
|  | Other stimulant disorders | Other stimulant disorders (ICD-10 code: F15) | present/absent |
|  | Cannabis use, unspecified | Cannabis use, unspecified (ICD-10 code: F12.9) | present/absent |
|  | Cannabis abuse | Cannabis abuse (ICD-10 code: F12.1) | present/absent |
|  | Cannabis dependence | Cannabis dependence (ICD-10 code: F12.2) | present/absent |
|  | Other psychoactive substance related disorders | Other psychoactive substance related disorders (ICD-10 code: F19) | present/absent |
|  | Family history of mental and behavioral disorders | Family history of mental and behavioral disorders (ICD-10 code: Z81) | present/absent |
|  | Substance Abuse Treatment | Substance Abuse Treatment (HCPCS Code: H) | present/absent |
|  | Psychotherapy Services and Procedures | Psychotherapy Services and Procedures (CPT code: 1021137) | present/absent |
|  | zolpidem | zolpidem (RxNorm code: 39993) | present/absent |
|  | buspirone | buspirone (RxNorm code: 1827) | present/absent |
|  | gabapentin | gabapentin (RxNorm code: 25480) | present/absent |

**Table S2**: Characteristics of the Semaglutide cohort and the non-GLP-1RA anti-obesity medications cohort for the study population with obesity who had a prior history of CUD (a recorded CUD diagnosis.

|  | **Before Propensity-Score Matching** | | | **After Propensity-Score Matching** | | |
| --- | --- | --- | --- | --- | --- | --- |
|  | **Semaglutide cohort** | **Non-GLP-1RA anti-obesity medications cohort** | **SMD** | **Semaglutide cohort** | **Non-GLP-1RA anti-obesity medications cohort** | **SMD** |
| **Total number** | 688 | 1,346 |  | 504 | 504 |  |
| **Age at index event (years, mean±SD)** | 47.6 ± 12.8 | 43.1 ± 12.9 | 0.35* | 46.1 ± 12.5 | 46.1 ± 12.8 | 0.002 |
| **Sex (%)** |  |  |  |  |  |  |
| Female | 54.4 | 53.4 | 0.02 | 56.9 | 57.9 | 0.02 |
| Male | 40.4 | 41.8 | 0.03 | 38.1 | 36.7 | 0.03 |
| Unknown | 5.2 | 4.8 | 0.02 | 5.0 | 5.4 | 0.02 |
| **Ethnicity (%)** |  |  |  |  |  |  |
| Hispanic/Latinx | 6.8 | 5.9 | 0.04 | 6.3 | 7.1 | 0.03 |
| Not Hispanic/Latinx | 76.3 | 77.4 | 0.03 | 78.0 | 76.4 | 0.04 |
| Unknown | 16.9 | 16.6 | 0.006 | 15.7 | 16.5 | 0.02 |
| **Race (%)** |  |  |  |  |  |  |
| Asian | 2.2 | 0.7 | 0.12* | 2.0 | 2.0 | <.001 |
| Black | 26.0 | 25.3 | 0.02 | 26.8 | 26.0 | 0.02 |
| White | 54.2 | 55.2 | 0.02 | 54.8 | 54.6 | 0.004 |
| Unknown | 11.8 | 13.5 | 0.05 | 11.1 | 12.5 | 0.04 |
| **Marital status (%)** |  |  |  |  |  |  |
| Never Married | 22.7 | 27.0 | 0.10* | 22.4 | 19.6 | 0.07 |
| Divorced | 6.7 | 6.0 | 0.03 | 6.3 | 6.3 | <.001 |
| Widowed | 1.5 | 1.5 | 0.003 | 2.0 | 2.0 | <.001 |
| **Adverse socioeconomic determinants of health (%)** | 17.7 | 28.8 | 0.27* | 18.5 | 17.5 | 0.03 |
| **Problems related to lifestyle (%)** | 34.2 | 37.7 | 0.08 | 37.3 | 34.5 | 0.06 |
| **Obesity categories (%)** |  |  |  |  |  |  |
| Morbid (severe) obesity due to excess calories | 71.7 | 45.5 | 0.55* | 66.5 | 70.4 | 0.09 |
| Obesity, unspecified | 80.1 | 75.6 | 0.11* | 79.0 | 78.6 | 0.01 |
| Other obesity due to excess calories | 18.2 | 10.4 | 0.22* | 14.7 | 14.3 | 0.01 |
| BMI 30.0-30.9 | 6.8 | 12.3 | 0.19* | 7.5 | 7.5 | <.001 |
| BMI 31.0-31.9 | 7.7 | 13.4 | 0.19* | 8.5 | 7.1 | 0.05 |
| BMI 32.0-32.9 | 9.6 | 11.7 | 0.07 | 9.7 | 8.7 | 0.03 |
| BMI 33.0-33.9 | 10.3 | 11.7 | 0.05 | 10.3 | 8.5 | 0.06 |
| BMI 34.0-34.9 | 10.2 | 11.8 | 0.05 | 10.9 | 10.5 | 0.01 |
| BMI 35.0-35.9 | 11.5 | 13.0 | 0.05 | 11.7 | 11.3 | 0.01 |
| BMI 36.0-36.9 | 12.8 | 11.4 | 0.04 | 11.3 | 11.5 | 0.006 |
| BMI 37.0-37.9 | 11.3 | 9.3 | 0.07 | 9.9 | 10.7 | 0.03 |
| BMI 38.0-38.9 | 13.2 | 9.1 | 0.13* | 12.1 | 11.7 | 0.01 |
| BMI 39.0-39.9 | 11.5 | 8.2 | 0.11* | 8.9 | 10.3 | 0.05 |
| BMI 40.0-44.9 | 30.1 | 22.1 | 0.18* | 27.8 | 29.4 | 0.04 |
| BMI 45.0-49.9 | 20.5 | 12.9 | 0.21* | 18.7 | 18.1 | 0.02 |
| BMI 50.0-59.9 | 15.6 | 10.1 | 0.16* | 14.9 | 14.1 | 0.02 |
| BMI 60.0-69.9 | 4.7 | 2.2 | 0.13* | 2.8 | 4.0 | 0.07 |
| BMI ≥70 | 2.9 | 0.8 | 0.16* | 2.0 | 2.0 | <.001 |
| **Family history of mental and behavioral disorders** | 4.9 | 9.3 | 0.17* | 4.4 | 3.8 | 0.03 |
| **Pre-existing medical conditions, procedures, medications (%)** |  |  |  |  |  |  |
| Type 2 diabetes | 64.2 | 29.6 | 0.74* | 55.0 | 54.0 | 0.02 |
| Depression | 61.5 | 65.8 | 0.09 | 61.7 | 62.1 | 0.008 |
| Mood disorders | 70.6 | 78.5 | 0.18* | 72.2 | 75.2 | 0.07 |
| Anxiety disorders | 67.0 | 77.5 | 0.24* | 69.6 | 72.4 | 0.06 |
| Psychotic disorders | 9.2 | 16.8 | 0.23* | 9.1 | 8.9 | 0.007 |
| Behavioral disorders | 17.2 | 15.2 | 0.05 | 17.9 | 16.1 | 0.05 |
| Disorders of adult personality and behavior | 11.6 | 13.1 | 0.04 | 10.9 | 10.7 | 0.006 |
| Behavioral and emotional disorders with onset usually occurring in childhood and adolescence | 12.4 | 14.3 | 0.06 | 12.1 | 12.7 | 0.02 |
| Conduct disorders | 2.2 | 3.6 | 0.09 | 2.4 | 2.2 | 0.01 |
| Symptoms and signs involving emotional state | 21.8 | 34.9 | 0.29* | 22.6 | 23.8 | 0.03 |
| Chronic pain | 53.5 | 42.9 | 0.21* | 49.6 | 48.6 | 0.02 |
| Cancer | 41.1 | 30.1 | 0.23* | 38.5 | 38.5 | <.001 |
| Alcohol use disorder | 23.7 | 33.2 | 0.21* | 23.8 | 23.8 | <.001 |
| Opioid use disorder | 16.6 | 18.1 | 0.04 | 15.3 | 15.7 | 0.01 |
| Tobacco use disorder | 58.9 | 67.7 | 0.18* | 62.5 | 60.1 | 0.05 |
| Cocaine use disorder | 15.0 | 16.9 | 0.05 | 13.9 | 11.7 | 0.07 |
| Other stimulant disorders | 12.4 | 15.2 | 0.08 | 11.5 | 10.7 | 0.03 |
| Other psychoactive substance related disorders | 21.9 | 28.0 | 0.14* | 22.0 | 20.4 | 0.04 |
| Hypertension | 80.7 | 72.2 | 0.20* | 77.6 | 77.6 | <.001 |
| Disorders of lipoprotein metabolism and other lipidemias | 73.0 | 50.1 | 0.48* | 67.7 | 65.7 | 0.04 |
| Hyperlipidemia | 61.2 | 39.9 | 0.44* | 55.8 | 52.2 | 0.07 |
| Hypercholesterolemia | 24.4 | 12.6 | 0.31* | 21.4 | 19.2 | 0.05 |
| Ischemic heart diseases | 29.2 | 21.3 | 0.18* | 27.2 | 24.0 | 0.07 |
| Other forms of heart disease | 50.3 | 46.1 | 0.08 | 46.4 | 47.4 | 0.02 |
| Cerebral infarction | 6.7 | 7.0 | 0.01 | 6.3 | 6.5 | 0.008 |
| Cerebrovascular diseases | 13.4 | 12.6 | 0.02 | 12.3 | 12.7 | 0.01 |
| Epilepsy and recurrent seizures | 4.8 | 8.9 | 0.16* | 6.0 | 4.8 | 0.05 |
| Migraine | 21.4 | 20.9 | 0.01 | 20.6 | 20.0 | 0.02 |
| Post-traumatic stress disorder (PTSD) | 15.0 | 22.1 | 0.19* | 15.7 | 14.9 | 0.02 |
| Substance abuse treatment | 1.5 | 5.6 | 0.23* | 2.0 | 2.0 | <.001 |
| Psychotherapy | 22.4 | 16.3 | 0.16* | 20.0 | 20.0 | <.001 |
| Zolpidem | 18.0 | 11.2 | 0.19* | 15.5 | 14.5 | 0.03 |
| Buspirone | 13.8 | 14.6 | 0.02 | 13.3 | 12.9 | 0.01 |
| Gabapentin | 46.4 | 39.7 | 0.14* | 45.0 | 42.9 | 0.04 |

Shown were cohorts before and after propensity-score matching for the listed variables with their status based on the presence of related clinical codes anytime to the day of the index event. SMD – standardized mean differences. *SMD greater than 0.1, a threshold indicating cohort imbalance. SD – standard deviation. Shown were cohorts before and after propensity-score matching for the listed variables with their status based on the presence of related clinical codes anytime on or before the index event (the first prescription of semaglutide, or non-GLP-1RA anti-obesity medications during 6/2021-12/2022). *SMD greater than 0.1, a threshold indicating cohort imbalance. Adverse socioeconomic determinants of health include problems related to education and literacy, employment and unemployment, housing and economic circumstances, social environment, upbringing, primary support group including family circumstances and various psychosocial circumstances. Problems with lifestyle included tobacco use, lack of physical exercise, inappropriate diet and eating habits, high-risk sexual behavior, gambling and betting, and other problems related to lifestyle including antisocial behavior and sleep deprivation.

**Table S3**: Characteristics of the Semaglutide cohort and the non-GLP-1RA anti-diabetes medications cohort for the study population with T2D who had a prior history of CUD (a recorded CUD diagnosis.

|  | **Before Propensity-Score Matching** | | | **After Propensity-Score Matching** | | |
| --- | --- | --- | --- | --- | --- | --- |
|  | **Semaglutide cohort** | **Non-GLP-1RA anti-diabetes medications cohort** | **SMD** | **Semaglutide cohort** | **Non-GLP-1RA anti-diabetes medications cohort** | **SMD** |
| **Total number** | 254 | 7,942 |  | 241 | 241 |  |
| **Age at index event (years, mean±SD)** | 52.7 ± 11.3 | 51.8 ± 12.2 | 0.07 | 52.4 ± 11.2 | 51.2 ± 12.4 | 0.09 |
| **Sex (%)** |  |  |  |  |  |  |
| Female | 40.2 | 32.3 | 0.16* | 39.4 | 38.6 | 0.02 |
| Male | 53.1 | 64.8 | 0.24* | 54.8 | 54.8 | <.001 |
| Unknown | 6.7 | 2.9 | 0.18* | 5.8 | 6.6 | 0.03 |
| **Ethnicity (%)** |  |  |  |  |  |  |
| Hispanic/Latinx | 6.3 | 5.3 | 0.04 | 6.2 | 5.4 | 0.04 |
| Not Hispanic/Latinx | 72.0 | 58.0 | 0.30* | 72.6 | 74.3 | 0.04 |
| Unknown | 21.7 | 36.7 | 0.34* | 21.2 | 20.3 | 0.02 |
| **Race (%)** |  |  |  |  |  |  |
| Asian | 3.9 | 2.0 | 0.11* | 4.1 | 4.1 | <.001 |
| Black | 31.9 | 22.9 | 0.04 | 30.7 | 34.4 | 0.08 |
| White | 44.5 | 47.4 | 0.06 | 46.5 | 42.3 | 0.08 |
| Unknown | 14.6 | 12.6 | 0.06 | 14.1 | 14.9 | 0.02 |
| **Marital status (%)** |  |  |  |  |  |  |
| Never Married | 17.7 | 19.1 | 0.03 | 17.0 | 20.7 | 0.09 |
| Divorced | 5.9 | 5.5 | 0.02 | 5.4 | 4.1 | 0.06 |
| Widowed | 3.9 | 1.9 | 0.12* | 4.1 | 4.1 | <.001 |
| **Adverse socioeconomic determinants of health (%)** | 15.4 | 14.5 | 0.02 | 14.9 | 17.8 | 0.08 |
| **Problems related to lifestyle (%)** | 30.7 | 18.4 | 0.29* | 30.3 | 35.3 | 0.11* |
| **Family history of mental and behavioral disorders** | 4.3 | 2.9 | 0.08 | 4.1 | 4.1 | <.001 |
| **Pre-existing medical conditions, procedures, medications (%)** |  |  |  |  |  |  |
| Depression | 50.8 | 31.5 | 0.40* | 49.8 | 49.4 | 0.008 |
| Mood disorders | 59.1 | 41.7 | 0.35* | 57.3 | 57.7 | 0.008 |
| Anxiety disorders | 60.2 | 38.6 | 0.44* | 58.9 | 55.6 | 0.07 |
| Psychotic disorders | 11.4 | 13.0 | 0.05 | 11.2 | 13.3 | 0.06 |
| Behavioral disorders | 16.1 | 3.6 | 0.43* | 15.4 | 16.6 | 0.03 |
| Disorders of adult personality and behavior | 7.9 | 5.2 | 0.11* | 8.3 | 11.2 | 0.09 |
| Behavioral and emotional disorders with onset usually occurring in childhood and adolescence | 7.1 | 3.7 | 0.15* | 7.1 | 7.5 | 0.02 |
| Conduct disorders | 3.9 | 1.2 | 0.17* | 4.1 | 4.1 | <.001 |
| Symptoms and signs involving emotional state | 18.5 | 15.5 | 0.08 | 18.3 | 16.2 | 0.06 |
| Chronic pain | 45.7 | 24.8 | 0.45* | 44.8 | 45.6 | 0.02 |
| Cancer | 42.9 | 18.3 | 0.56* | 41.1 | 39.0 | 0.04 |
| Alcohol use disorder | 23.6 | 25.5 | 0.04 | 24.1 | 19.9 | 0.10* |
| Opioid use disorder | 16.9 | 11.5 | 0.08 | 16.2 | 17.8 | 0.04 |
| Tobacco use disorder | 54.7 | 60.5 | 0.12* | 55.6 | 57.3 | 0.03 |
| Cocaine use disorder | 16.5 | 18.5 | 0.05 | 16.2 | 15.8 | 0.01 |
| Other stimulant disorders | 11.0 | 12.0 | 0.03 | 10.8 | 11.6 | 0.03 |
| Other psychoactive substance related disorders | 19.3 | 17.1 | 0.06 | 19.5 | 19.5 | <.001 |
| Hypertension | 92.5 | 87.8 | 0.16* | 92.1 | 94.6 | 0.10* |
| Disorders of lipoprotein metabolism and other lipidemias | 87.0 | 55.1 | 0.75* | 86.3 | 85.5 | 0.02 |
| Hyperlipidemia | 78.0 | 46.7 | 0.68* | 76.8 | 75.1 | 0.04 |
| Hypercholesterolemia | 33.1 | 16.1 | 0.40* | 32.1 | 28.2 | 0.11* |
| Ischemic heart diseases | 37.4 | 30.9 | 0.14* | 37.3 | 36.1 | 0.03 |
| Other forms of heart disease | 55.9 | 41.2 | 0.30* | 54.8 | 51.0 | 0.08 |
| Cerebral infarction | 11.0 | 7.7 | 0.11* | 10.4 | 10.8 | 0.01 |
| Cerebrovascular diseases | 20.5 | 13.7 | 0.18* | 19.1 | 17.8 | 0.03 |
| Epilepsy and recurrent seizures | 6.7 | 6.9 | 0.007 | 6.6 | 7.5 | 0.03 |
| Migraine | 15.7 | 6.2 | 0.31* | 15.8 | 14.5 | 0.04 |
| Post-traumatic stress disorder (PTSD) | 9.1 | 8.7 | 0.01 | 8.7 | 10.0 | 0.04 |
| Morbid (severe) obesity due to excess calories | 45.7 | 15.7 | 0.69* | 43.6 | 51.0 | 0.15* |
| Obesity, unspecified | 65.7 | 26.4 | 0.86* | 64.3 | 63.9 | 0.009 |
| Other obesity due to excess calories | 11.8 | 1.8 | 0.41* | 10.0 | 10.8 | 0.03 |
| BMI 30.0-30.9 | 7.9 | 2.9 | 0.22* | 7.1 | 5.8 | 0.05 |
| BMI 31.0-31.9 | 13.0 | 2.9 | 0.38* | 11.6 | 10.4 | 0.04 |
| BMI 32.0-32.9 | 7.9 | 2.9 | 0.22* | 7.1 | 5.0 | 0.09 |
| BMI 33.0-33.9 | 7.1 | 2.9 | 0.20* | 6.2 | 5.0 | 0.05 |
| BMI 34.0-34.9 | 10.2 | 2.6 | 0.32* | 8.7 | 7.9 | 0.03 |
| BMI 35.0-35.9 | 11.0 | 2.6 | 0.34* | 8.3 | 7.1 | 0.05 |
| BMI 36.0-36.9 | 7.9 | 2.5 | 0.25* | 6.2 | 4.6 | 0.07 |
| BMI 37.0-37.9 | 10.2 | 2.5 | 0.25* | 9.5 | 10.4 | 0.03 |
| BMI 38.0-38.9 | 9.1 | 2.3 | 0.30* | 8.7 | 7.5 | 0.05 |
| BMI 39.0-39.9 | 9.4 | 2.0 | 0.32* | 8.7 | 7.3 | 0.02 |
| BMI 40.0-44.9 | 20.5 | 6.7 | 0.41* | 19.1 | 17.8 | 0.03 |
| BMI 45.0-49.9 | 12.6 | 3.5 | 0.34* | 11.6 | 15.8 | 0.12* |
| BMI 50.0-59.9 | 8.7 | 2.8 | 0.26* | 8.3 | 9.1 | 0.03 |
| BMI 60.0-69.9 | 3.9 | 0.5 | 0.24* | 4.1 | 4.1 | <.001 |
| BMI ≥70 | 3.9 | 0.5 | 0.24* | 4.1 | 4.1 | <.001 |
| Substance abuse treatment | 3.9 | 3.2 | 0.04 | 4.1 | 4.1 | <.001 |
| Psychotherapy | 11.4 | 4.1 | 0.28* | 11.2 | 9.1 | 0.07 |
| Zolpidem | 18.9 | 6.7 | 0.37* | 17.8 | 16.6 | 0.03 |
| Buspirone | 7.1 | 3.6 | 0.16* | 6.6 | 5.4 | 0.05 |
| Gabapentin | 52.0 | 22.6 | 0.64* | 49.8 | 49.8 | <.001 |

Shown were cohorts before and after propensity-score matching for the listed variables with their status based on the presence of related clinical codes anytime to the day of the index event. SMD – standardized mean differences. *SMD greater than 0.1, a threshold indicating cohort imbalance. SD – standard deviation. Shown were cohorts before and after propensity-score matching for the listed variables with their status based on the presence of related clinical codes anytime on or before the index event (the first prescription of semaglutide, or non-GLP-1RA anti-diabetes medications during 12/2017-5/2021). *SMD greater than 0.1, a threshold indicating cohort imbalance. Adverse socioeconomic determinants of health include problems related to education and literacy, employment and unemployment, housing and economic circumstances, social environment, upbringing, primary support group including family circumstances and various psychosocial circumstances. Problems with lifestyle included tobacco use, lack of physical exercise, inappropriate diet and eating habits, high-risk sexual behavior, gambling and betting, and other problems related to lifestyle including antisocial behavior and sleep deprivation.
